# Mondor’s Disease: A Rare Cause of Chest Pain

**DOI:** 10.7759/cureus.22320

**Published:** 2022-02-17

**Authors:** Dharmendra K Pipal, Vibha Rani Pipal

**Affiliations:** 1 General Surgery, All India Institute of Medical Sciences, Gorakhpur, Gorakhpur, IND; 2 Obstetrics and Gynecology, All India Institute of Medical Sciences, Gorakhpur, Gorakhpur, IND

**Keywords:** lateral thoracic vein, carcinoma breast, thrombophlebitis, mondor’s disease, chest pain

## Abstract

Mondor’s disease is an uncommon cause of breast and chest pain. It is characterized by cord-like thickening of the superficial veins of the anterior chest wall mimicking many life-threatening illnesses such as pulmonary thromboembolism and myocardial infarction. The disease may have been caused by trauma, other hypercoagulable states, or underlying breast diseases such as infection or carcinoma breast, but, in most cases, its etiology remains unknown. Mondor’s disease is usually self-limited and can be managed conservatively. Although a rare and benign diagnosis, Mondor’s disease should be a part of the differentials of chest pain, and its diagnosis can be made on the basis of a thorough clinical examination alone, which reduces not only costs but also the risks of further testing for patients presenting with chest pain.

We highlight the case of a 40-year-old premenopausal female patient who presented to the outpatient department with stretching aching chest pain on the left side, which got aggravated on movements of the arm and relieved on rest.

Mondor’s disease is not considered a differential diagnosis for chest pain due to a lack of awareness about it. Creating awareness of this condition through this case report would help to reduce unnecessary investigations and valuable time spent and would help identify a serious underlying cause, especially early stage carcinoma of the breast.

## Introduction

Henri Mondor, a French surgeon, published a case series in 1939 where he described Mondor's disease in detail [1]. It is a rare benign disorder of the breast and is characterized by chest wall thrombophlebitis. In 50-60% of cases, the disease is idiopathic (primary disease), while approximately 40%-50% of cases are associated with some inciting factors such as trauma or underlying malignancy [2].

With the rarity of the disease as well as the absence of definitive diagnostic criteria, Mondor's disease is frequently underdiagnosed. The majority of cases present with cord-like thickening over the chest wall or breast due to thrombosis of the superficial veins, and a few may present with chest pain due to thrombophlebitis. Males can present as cases of penile Mondor's disease. Clinically, it manifests as a cord-like induration on the chest wall and resolves spontaneously without any sequela. Although it is most frequently identified in the chest wall, it has been described in other locations as well, such as the groin, penis, and antecubital fossa [3].

## Case presentation

History

A 40-year-old premenopausal female presented with complaints of stretching pain in her left chest wall for 10 days. She had taken some analgesic, which relieved the symptoms to some extent, but soon she noticed a cord-like thickening on the left side of the chest wall. On elaborating her history, she revealed that she had the same symptoms but the swelling was located in the left axilla one year ago, and it was relieved by antibiotics and anti-inflammatory drugs prescribed by a physician. She denied symptoms related to pleurisy, such as fever, cough, shortness of breath, headache, or rash. She denied any recent history of surgery or trauma. In menstrual history, the cycle was regular, lasting for four to five days, and had last menstruation seven days ago the day of presentation. Personal and family history was negative with regard to secondary hypercoagulable states such as malignancy, pregnancy, use of oral contraceptives, infusion of prothrombin complex concentrates, and autoimmune coagulopathies such as systemic lupus erythematosus or inflammatory bowel disease.

Examination findings

Her blood pressure was 130/80 mmHg, heart rate was 80 beats per minute, temperature was 36.2°C, respiratory rate was 20 breaths per minute, and SpO_2_ was 99% on room air. She was alert and oriented, and on auscultation, bilateral air entry and heart sounds were normal.

**Figure 1:**
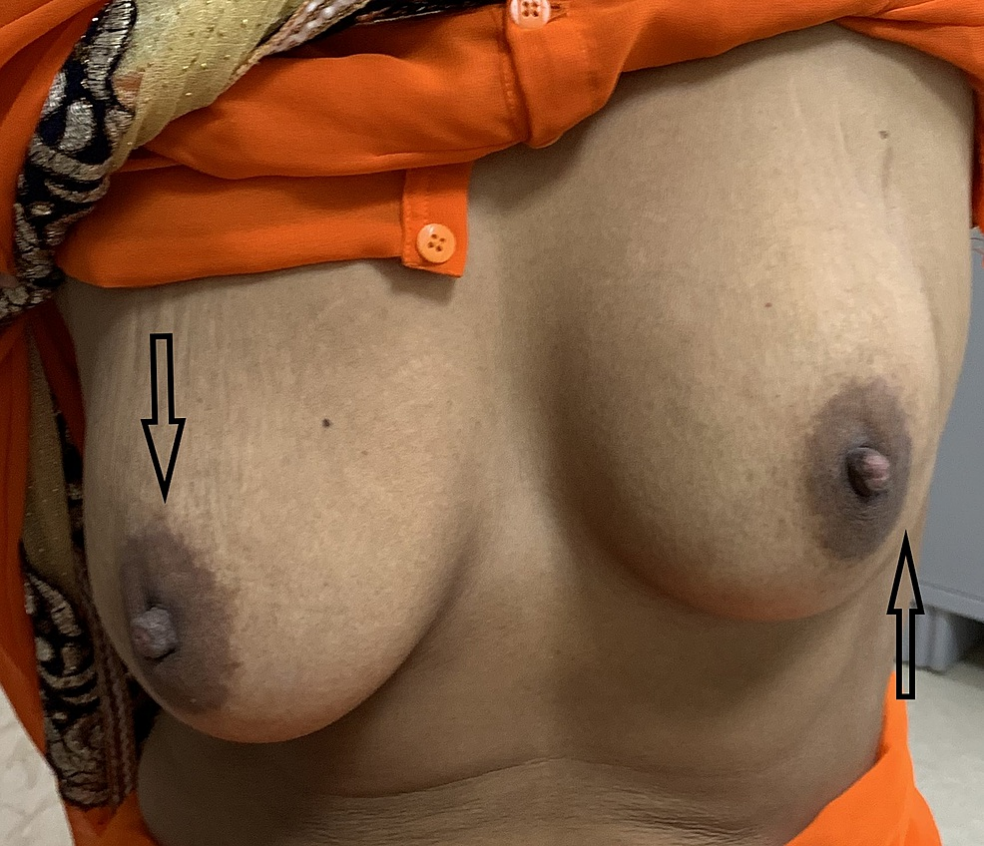
Left breast drew up on raising the arms

**Figure 2:**
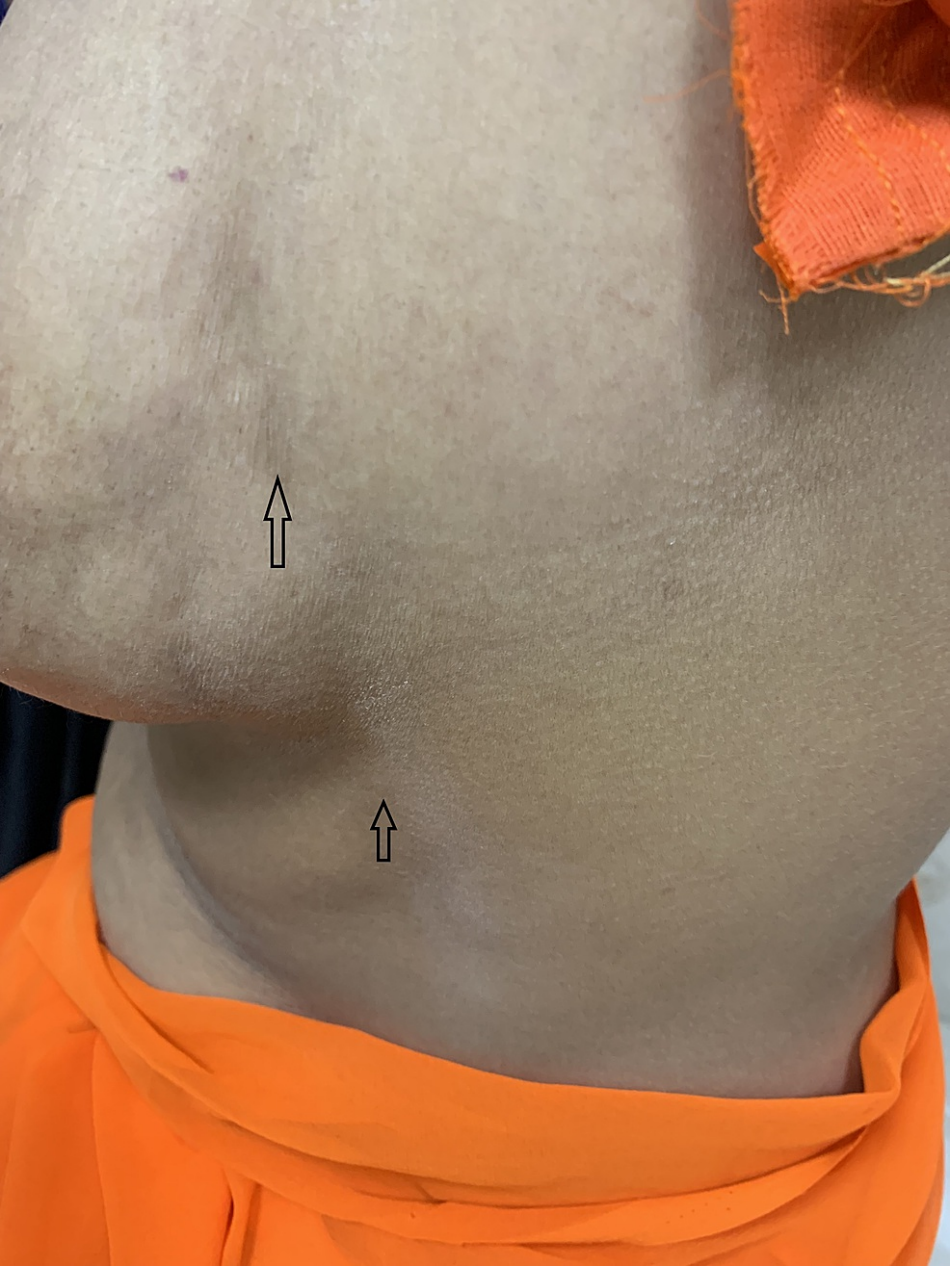
Depression at the site of thrombophlebitis over breast along with thick cord of thrombosed vein on the chest wall

On examination, there was a thickened cord-like structure extending from the left lower chest to the upper outer quadrant of the breast. The breast was normal on examination, but on raising the hand above the head, the left breast was pulled upward (Figure 1) along with the appearance of a shallow vertical depression along the course of the affected vein (Figure 2). On palpation, a thickened, tender, and mobile cord of the thrombosed vein was palpable. Breast parenchyma, axilla, and upper arm were normal. All other systemic examinations were grossly normal.

Diagnostic evaluation

**Figure 3:**
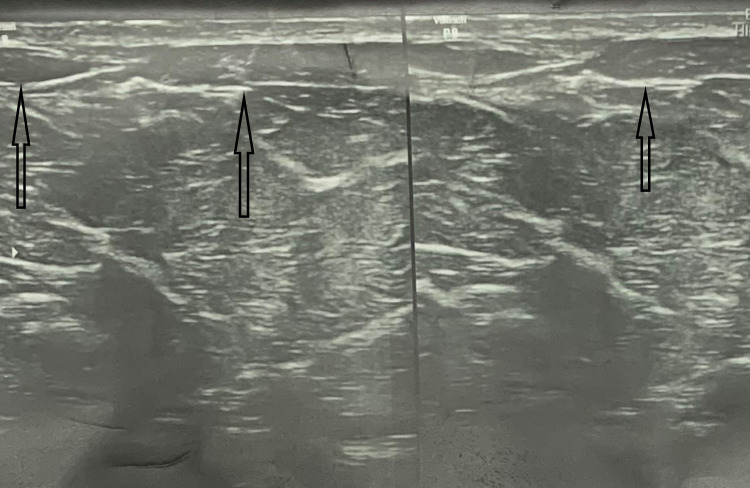
Ultrasound showing dilated superficial vein of the chest wall and breast

Blood biochemistry included total blood count, platelet count, erythrocyte sedimentation rate, and liver function with prothrombin time and international normalized ratio (PT-INR). Renal function and ECG were normal. Ultrasonographic findings recorded a non-compressible hypoechoic left lateral thoracic vein (Figure 3). No deep vein thrombosis was observed clinically as well as on the Doppler study. Both the breast and axilla were normal on ultrasonography as well as on mammographic evaluation. Following a course of anti-inflammatory drug, diclofenac, both oral and topical, the patient was followed up for a month, and she was recovered clinically.

 

## Discussion

The most common veins that can be affected are thoracoepigastric, lateral thoracic, and superficial epigastric veins. Penile Mondor's disease is thrombosis of the dorsal superficial vein of the penis; it was reported by Helm and Hodge in 1958 [4]. In our patient, the lateral thoracic vein was affected. Most commonly, the disease is idiopathic, but other etiological factors including hormone therapy, breast cancer, thrombophilic conditions, coagulopathies, and surgical or physical trauma have also been identified [5].

With elaborated history, clinical examination, and investigations, we did not find any risk factors in our patient. A palpable tender cord-like structure that relates to a superficial vein and chest pain are the usual presentations of Mondor's disease [6].

However, the pathophysiology of this condition is unclear, but various mechanisms such as direct trauma and stagnation of blood in veins may play a key role in the development of the disease [7]. Duplex scan described the thickened vein as a long, superficial, tubular, non-echoic cord-like structure with no blood flow. Those who have a palpable lump in the breast with thrombophlebitis require mammographic or ultrasonographic evaluation to exclude a malignant etiology [8].

In spite of its self-limiting nature, few cases require treatment such as topical or oral non-steroidal anti-inflammatory drugs such as diclofenac to get rid of the pain and swelling. Antimicrobials are required only in associated infections [9,10]. Systemic anticoagulation such as low molecular weight heparin or oral anticoagulants is recommended only for high-risk patients [11]. Pain and size of thrombus can be reduced with the help of oral anticoagulants [12]. Our patient recovered following a course of anti-inflammatory drugs within a month.

## Conclusions

Mondor's disease is rarely considered as a differential diagnosis in a patient with chest pain due to its rarity and lack of awareness. Sometimes, it may be the only manifestation of some underlying serious medical diseases such as myocardial infarction and pulmonary embolism, and once these are ruled out, a surgical disease, especially breast carcinoma, can present as Mondor's disease. Therefore, listing it as a differential diagnosis may play a crucial role in the early diagnosis and treatment of other serious conditions such as carcinoma of the breast. A thorough history and clinical examination and ECG are sometimes sufficient to diagnose the disease and could help in cost reduction by avoiding unnecessary investigations.
